# Patient With Penile and Scrotal Strangulation Due to Prolonged Use of a Metal Ring Device

**DOI:** 10.7759/cureus.11928

**Published:** 2020-12-05

**Authors:** Emily Harris, Diana Llompart, Guillermo Izquierdo, Muhammad A Aziz

**Affiliations:** 1 Internal Medicine, Florida International University, Herbert Wertheim College of Medicine, Miami, USA; 2 Internal Medicine, Jackson Memorial Hospital, Miami, USA

**Keywords:** penile entrapment, strangulation

## Abstract

Penile and scrotal incarceration by a metal ring is a rare urological emergency that requires immediate removal of the strangulating object to avoid severe clinical consequences. Metal rings are used to enhance sexual activity. Here we present a case of penile and scrotal entrapment in a young patient who presented three hours after removal with fever and pain. Before removal, the ring had remained in place for over 48 hours. This case highlights the importance of prompt treatment of these patients as complications such as gangrene can occur if not treated expediently.

## Introduction

Penile rings are used to enhance sexual function by constricting the outflow of blood from the corpus cavernosum, which prolongs erection. However, penile rings can lead to genital incarceration if left on for over 30 minutes [1]. Penile ring entrapment is considered a urological emergency with potentially severe clinical consequences, including death if left untreated [1,2].

Penile incarceration has been described in the literature through sporadic case studies and has affected a wide range of age groups. The most frequently used penile rings causing incarceration are metal rings, although non-metal rings have also been reported to cause an injury that is often more severe. Penoscrotal rings are used less frequently in sexual practices but can cause significant injury if entrapment occurs. Emergent removal is necessary, and delay in treatment may result in edema, tissue injury, necrosis or eventual gangrene [1].

## Case presentation

A 28-year-old man with a past medical history of HIV, hepatitis C (untreated), and secondary syphilis presented to the emergency department with complaints of new-onset fever, plus penile and testicular swelling for the past 72 hours. The previous day he had visited the emergency department because he was unable to remove a metal ring that he had placed around the base of his penis and scrotum two days earlier. He had been able to urinate with a normal urinary stream. The patient's past medical history included HIV diagnosed 2011, hepatitis C diagnosed in 2018, and secondary syphilis. The patient also had a history of tobacco and methamphetamine use. He was afebrile, tachycardic with a heart rate of 109 beats/minute, with a blood pressure of 141/115 mmHg. Attempts to pull off the ring with lubrication were futile, and the ER team subsequently split and removed the ring with an electric saw. The patient was discharged early in the morning with plans for urology follow up and sent home with a prescription for cephalexin 500 mg four times a day for seven days. 

**Figure 1:**
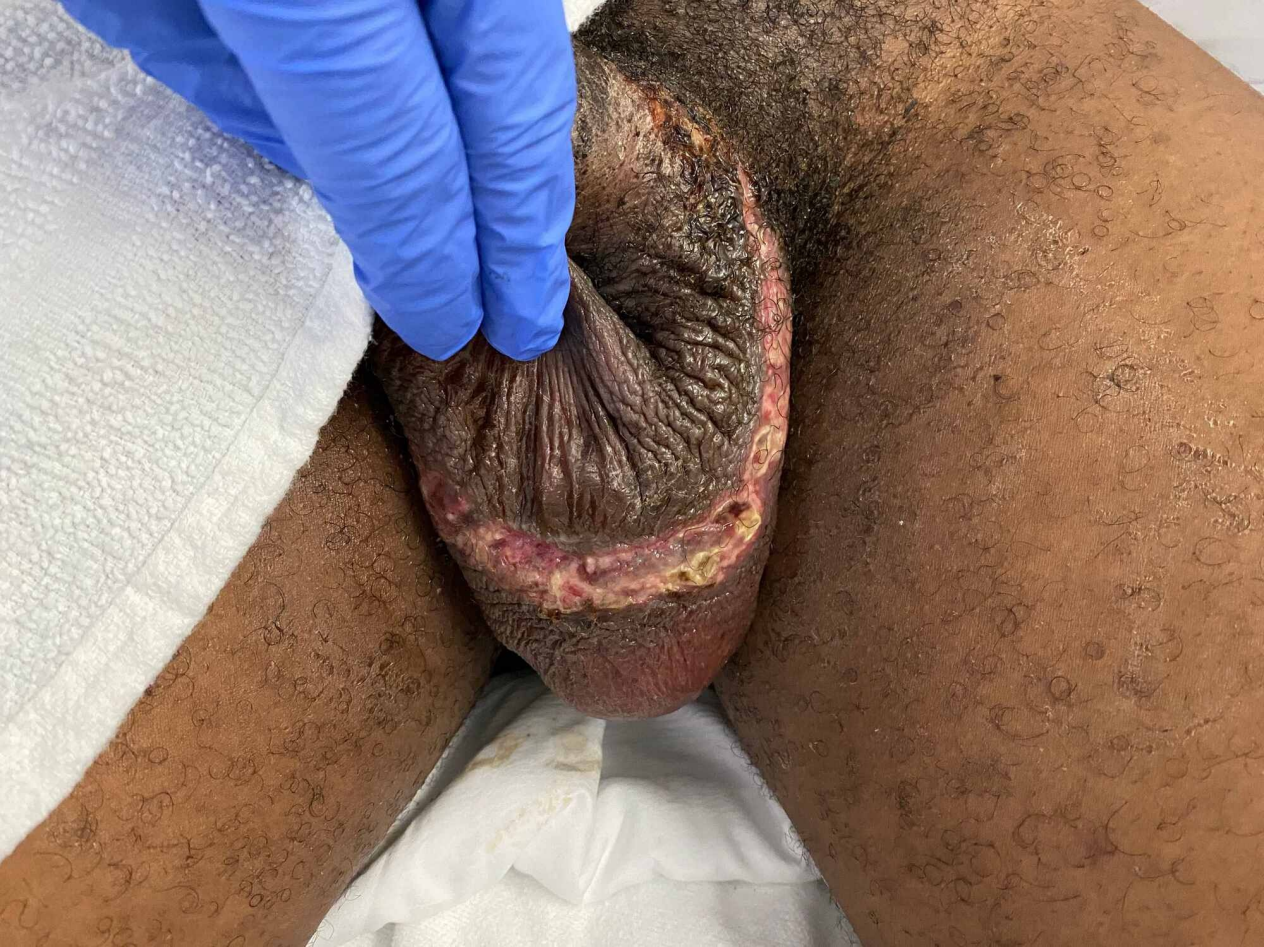
Shown is the resultant scrotal injury with destructive skin changes (friable tissue with ulceration and purulent drainage) where the constrictor had been placed

He returned three hours later, that same morning with complaints of a fever, sweats, nausea, chills, and muscle fatigue. He denied respiratory symptoms, dysuria, abdominal pain, nausea, vomiting. On physical examination, the patient appeared to be in moderate distress. Presenting vital signs were as follows: temperature 39.5°C, heart rate 126, respiratory rate 20 breaths per minute, blood pressure 142/68 mmHg, O2 saturation on room air of 98-100%. The genitourinary examination was significant for severe edema over the body of the penis and testicles, as seen in Figure 1. Over the area where the annular penile constrictor was placed, there was some skin maceration and minor purulent discharge. Sensation in the penile and scrotal area remained intact. There were no signs of surrounding cellulitis. Cardiac, respiratory, and abdominal examinations were unremarkable. The patient was admitted and started on intravenous cefepime and vancomycin along with topical bacitracin. The patient had a mildly elevated white blood cells count with neutrophilic predominance, normal lactic acid level. HIV antigen and antibody test was reactive with a CD4 count of 854. The patient's liver enzymes were moderately elevated, likely due to untreated hepatitis C. The rest of the patient's laboratory results are detailed in Table 1.

**Table 1:** Laboratory Values HCV- Hepatitis C virus

	Patient Values	Normal
White blood cells (k/mm^3^)	11.0	4-10.5
Neutrophils %	75.2	36-70
Lymphocytes %	14.9	16-43
Absolute Neutrophils (x10^3^/mm^3^)	8.3	1.5-8
Absolute Lymphocytes (x10^3^/mm^3^)	1.6	1-4
Hemoglobin (g/dL)	14.1	11.1-14.6
Hematocrit (g/dL)	42.9	33.2-43.4
Platelets (x10^3^/mcl)	269	140.0-400.0
Aspartate aminotransferase (U/L)	79	15-46
Alanine aminotransferase (U/L)	106	9-52
Alkaline phosphatase (U/L)	66	38-126
Lactic acid (mmol/L)	0.6	0.5-1
HCV	reactive	-
HIV Ag/Ab	reactive	-
CD4 count (cells/mm^3^)	854.92	500-1200

**Figure 2:**
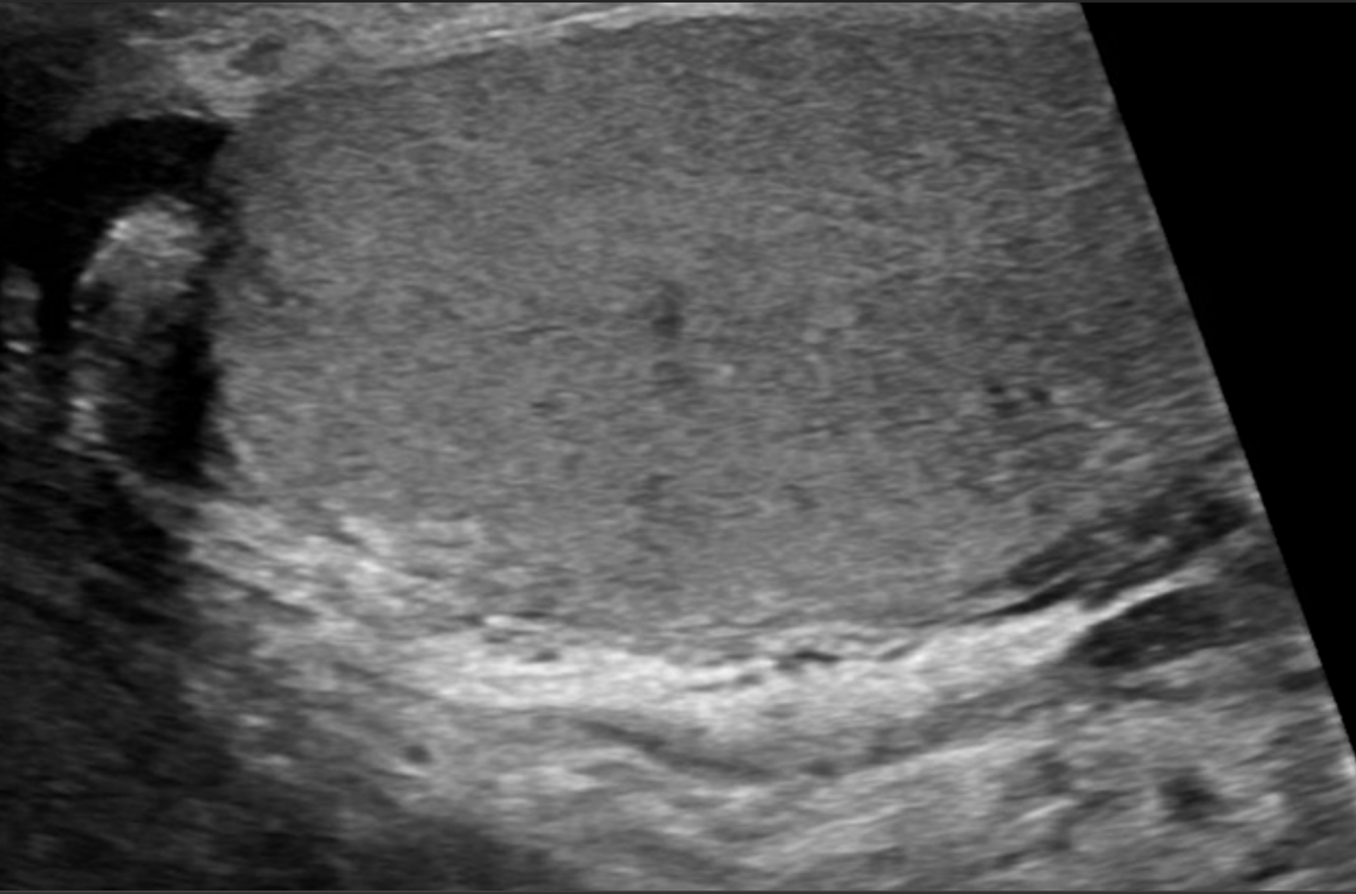
Ultrasound of scrotum & right testicle

Ultrasound of the scrotum with duplex showed increased vascularity of the enlarged left epididymal tail, concerning for epididymitis as seen in Figure 2. There was soft tissue swelling of the scrotum that was worse on the right. Additionally, mobile echoes within the edematous scrotum were seen for which an unencapsulated complex fluid collection such as abscess or hematoma could not be excluded. There was no sonographic evidence of soft tissue gas, ruling down the possibility of gas gangrene. Cultures of the wound revealed massive growth of two strains of gram-negative rods and heavy growth of gram-positive cocci.

Urology was consulted who recommended continuing broad-spectrum antibiotics with no surgical intervention recommended. The infectious disease team was also consulted, and they suggested to broaden antibiotic coverage to cover anaerobes by adding metronidazole. Over the next week, the patient made a full recovery with no residual sensory or functional deficits. He was discharged on oral and topical antibiotics.

## Discussion

Rings placed around the penis and scrotum are used to restrict blood flow to increase sexual performance. Penile entrapment related to excessive constriction of the tissue can occur, causing progressive edema, ischemia and if left untreated gangrene [1]. This case highlights the potential complications of penile ring entrapment in this young patient with delayed treatment. In cases involving penile rings, patients often delay seeking care due to the sensitive nature of their issue. There have been a few cases reported in the literature of patients with resultant sepsis from penile ring injury with a couple of reports resulting in the death of the patient [3,4]. 

In this case, the patient was lost to follow-up as he decided to continue his care in a separate community clinic. Due to loss to follow-up, we are limited in regards to discussing the outcomes for this patient.

Patients who present with a longer duration of entrapment and who use non-metal objects more frequently experience higher grade penile injuries with diminished recovery and less favourable outcomes [3]. Also, physicians often have to be resourceful when removing the penile ring, often relying on tools available in the maintenance department of their hospital, which may not be optimal.

This case emphasizes the importance of prompt treatment with removal of the ring and the initiation of broad-spectrum antibiotics in any patient with a similar localized injury sustained over days.

## Conclusions

Penile ring entrapment is a rare, urological emergency that can lead to severe consequences such as gangrene or even penile amputation if not promptly treated. A rapid removal of the constricting device is of the utmost importance and can require an inventive approach. While most patients do well without additional interventions, it is essential to initiate antibiotic treatment and closely monitor patients to prevent ischemia, necrosis, gangrene or systemic complications. Another critical takeaway point from this case is the emphasis on patient education regarding safe sexual practices.
